# Supplementary material for: Temporal epigenome modulation enables efficient bacteriophage engineering and functional analysis of phage DNA modifications
Source: PLoS Genet. 2024 Sep 4;20(9):e1011384. doi: 10.1371/journal.pgen.1011384 (PMC11404850; doi:10.1371/journal.pgen.1011384)
Supplement: S1 Fig — (A) Impact of NgTET recombinant expression on E. coli growth. n = 3 biological replicates. (B) Lysis of E. coli by the phages recovered from different conditions. n = 3 biological replicates. (PDF) [file pgen.1011384.s001.pdf]

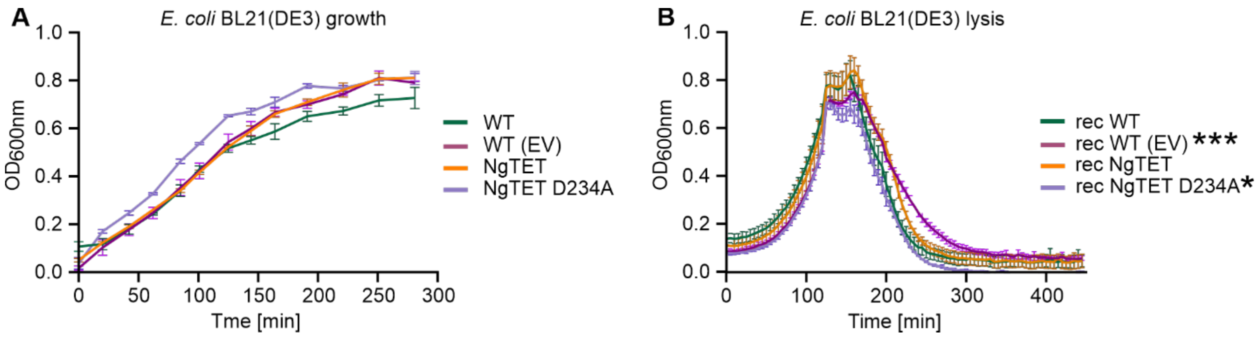

S1 Fig: Growth and lysis of *E. coli* upon different conditions.

(**A**) Impact of NgTET recombinant expression on *E. coli* growth. *n* = 3 biological replicates. (**B**) Lysis of *E. coli* by the phages recovered from different conditions. *n* = 3 biological replicates.
