# Supplementary material for: Temporal epigenome modulation enables efficient bacteriophage engineering and functional analysis of phage DNA modifications
Source: PLoS Genet. 2024 Sep 4;20(9):e1011384. doi: 10.1371/journal.pgen.1011384 (PMC11404850; doi:10.1371/journal.pgen.1011384)
Supplement: S2 Fig — (A-B) Extracted ion chromatogram overlay of nucleotide mass traces in T4 WT DNA (front) and NgTET-treated T4 DNA (back). Data exemplifies differences in relative abundances of cysteine nucleotides (dC, 5hmdC, 5fdC, 5cadC) (B) while dG, dT and dA (A) show similar trends. Signal intensities have been normalized against the overall nucleotide signal in the respective sample to correct for differences in sample concentration and injection volume. (C-D) Extracted ion chromatogram of presumed 5ghmdC (C). The fragmentation pattern of presumed 5ghmdC corresponds to the predicted one (D). (E) Relative abundance of cytosine derivatives in T4 phage isolated from different strains (two-sided Student´s t-test, *—Psignif < 0.05, **—Psignif < 0.025; n.d.: not detected). n = 3 biological replicates. (PDF) [file pgen.1011384.s002.pdf]

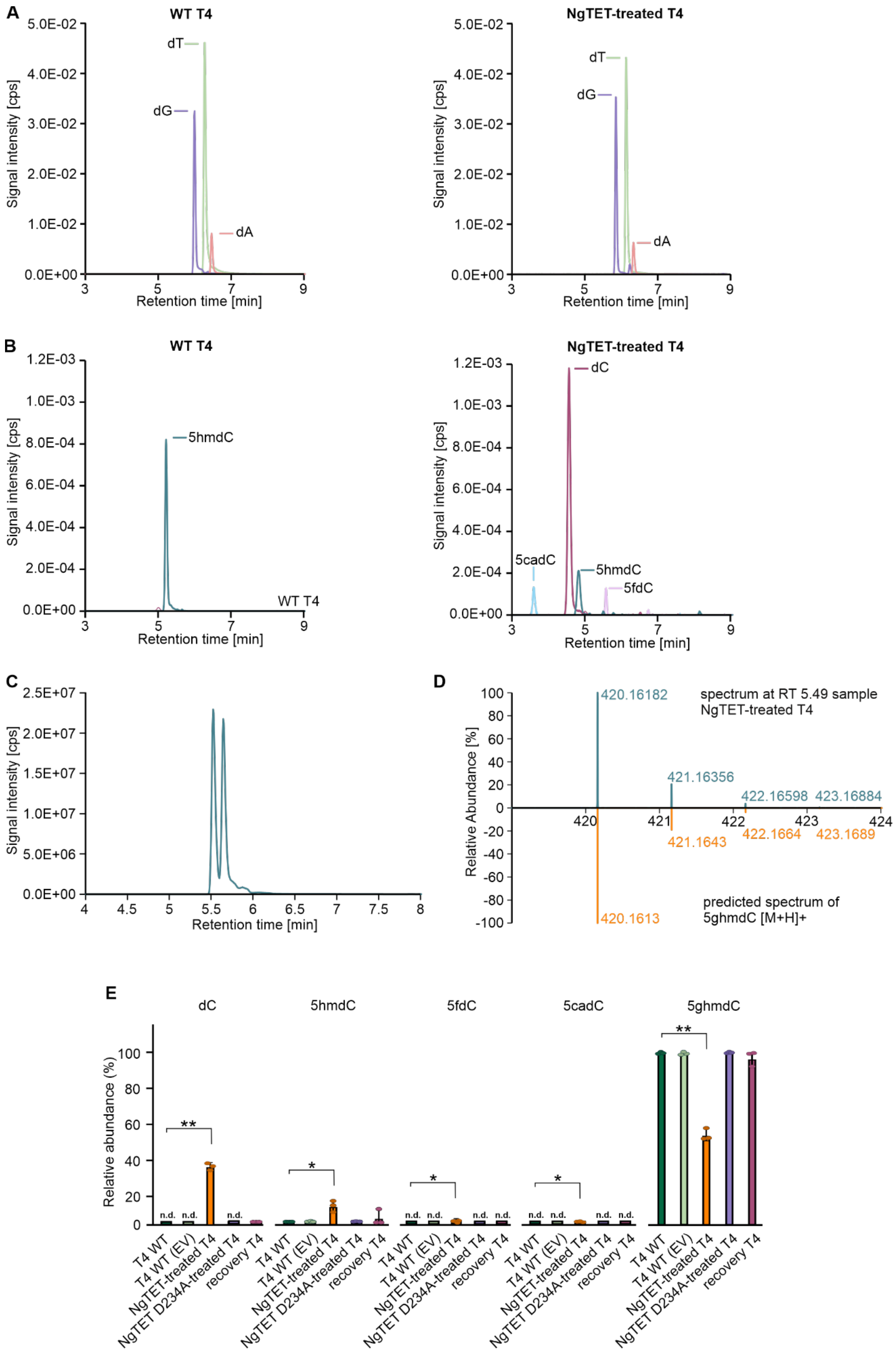

S2 Fig: LC-MS analysis of T4 DNA composition.

(**A-B**) Extracted ion chromatogram overlay of nucleotide mass traces in T4 WT DNA (front) and NgTET-treated T4 DNA (back). Data exemplifies differences in relative abundances of cysteine nucleotides (dC, 5hmdC, 5fdC, 5cadC) (**B**) while dG, dT and dA (**A**) show similar trends. Signal intensities have been normalized against the overall nucleotide signal in the respective sample to correct for differences in sample concentration and injection volume. (**C-D**) Extracted ion chromatogram of presumed 5ghmdC (**C**). The fragmentation pattern of presumed 5ghmdC corresponds to the predicted one (**D**). (**E**) Relative abundance of cytosine derivatives in T4 phage isolated from different strains (two-sided Student´s t-test, * - $P_{signif} < 0.05$, ** - $P_{signif} < 0.025$; n.d.: not detected). n = 3 biological replicates.
