# Supplementary material for: Temporal epigenome modulation enables efficient bacteriophage engineering and functional analysis of phage DNA modifications
Source: PLoS Genet. 2024 Sep 4;20(9):e1011384. doi: 10.1371/journal.pgen.1011384 (PMC11404850; doi:10.1371/journal.pgen.1011384)
Supplement: S3 Fig — (A) Impact of CRISPR/Cas9 or Cas12 systems recombinant expression on E. coli growth. n = 3 biological replicates. (B) Impact of CRISPR/Cas12 system recombinant expression on T4 WT and T4 NgTET lysis efficiency. n = 3 biological replicates. (PDF) [file pgen.1011384.s003.pdf]

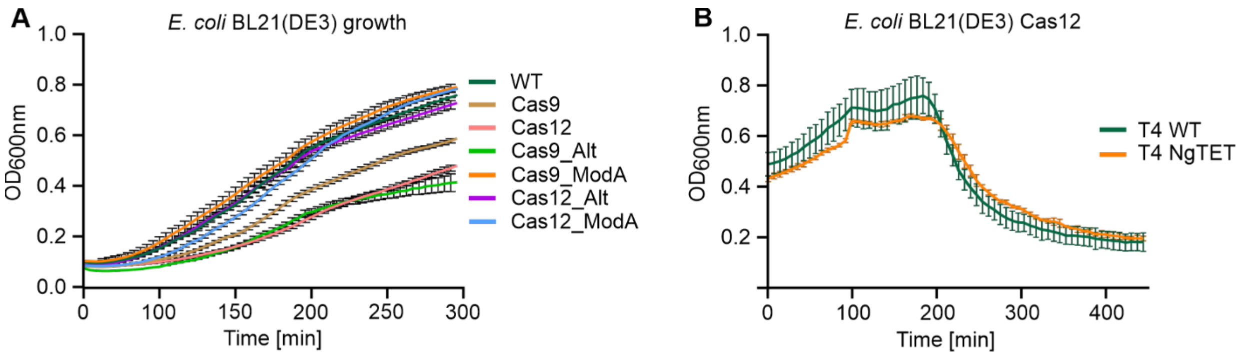

S3 Fig: Effect of Cas expression on growth and lysis of *E. coli*. (**A**) Impact of CRISPR/Cas9 or Cas12 systems recombinant expression on *E. coli* growth. *n* = 3 biological replicates. (**B**) Impact of CRISPR/Cas12 system recombinant expression on T4 WT and T4 NgTET lysis efficiency. *n* = 3 biological replicates.
