# Supplementary material for: Temporal epigenome modulation enables efficient bacteriophage engineering and functional analysis of phage DNA modifications
Source: PLoS Genet. 2024 Sep 4;20(9):e1011384. doi: 10.1371/journal.pgen.1011384 (PMC11404850; doi:10.1371/journal.pgen.1011384)
Supplement: S4 Fig — (A-B) Stain-free scan (TCE stain, loading control, left) and Western blot analysis (pan-ADPr antibody for ADP-ribosylation detection, right) to identify ADP-ribosylation events by ARTs and their mutants. Both mutants demonstrate complete abolishment of ADP-ribosylation. n = 3 biological replicates, a representative example is shown. (PDF) [file pgen.1011384.s004.pdf]

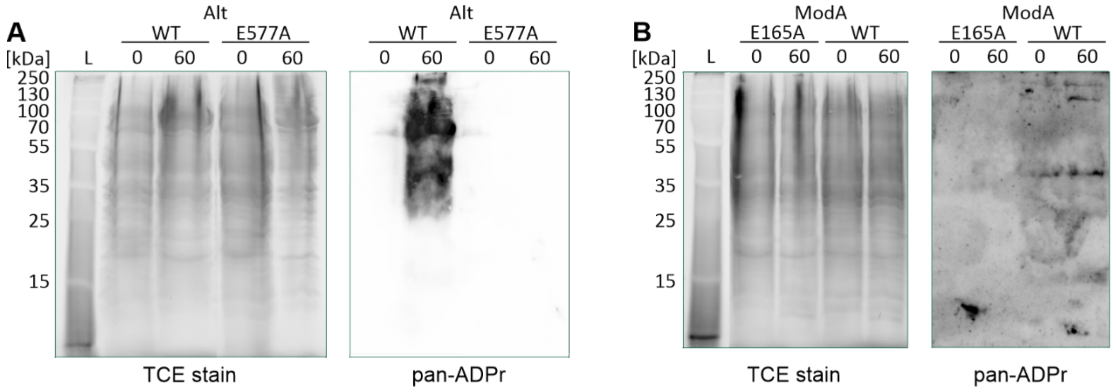

S4 Fig: Validation of Alt and ModA ARTs inactivation via Alt E577A and ModA E165A mutations. (**A-B**) Stain-free scan (TCE stain, loading control, left) and Western blot analysis (pan-ADPr antibody for ADP-ribosylation detection, right) to identify ADP-ribosylation events by ARTs and their mutants. Both mutants demonstrate complete abolishment of ADP-ribosylation. *n* = 3 biological replicates, a representative example is shown.
