# Supplementary material for: Temporal epigenome modulation enables efficient bacteriophage engineering and functional analysis of phage DNA modifications
Source: PLoS Genet. 2024 Sep 4;20(9):e1011384. doi: 10.1371/journal.pgen.1011384 (PMC11404850; doi:10.1371/journal.pgen.1011384)
Supplement: S5 Fig — (A) The targeted mutation site in alt gene. (B) Sequence coverage at the mutation site. (C): Alt E577A mutant. (PDF) [file pgen.1011384.s005.pdf]

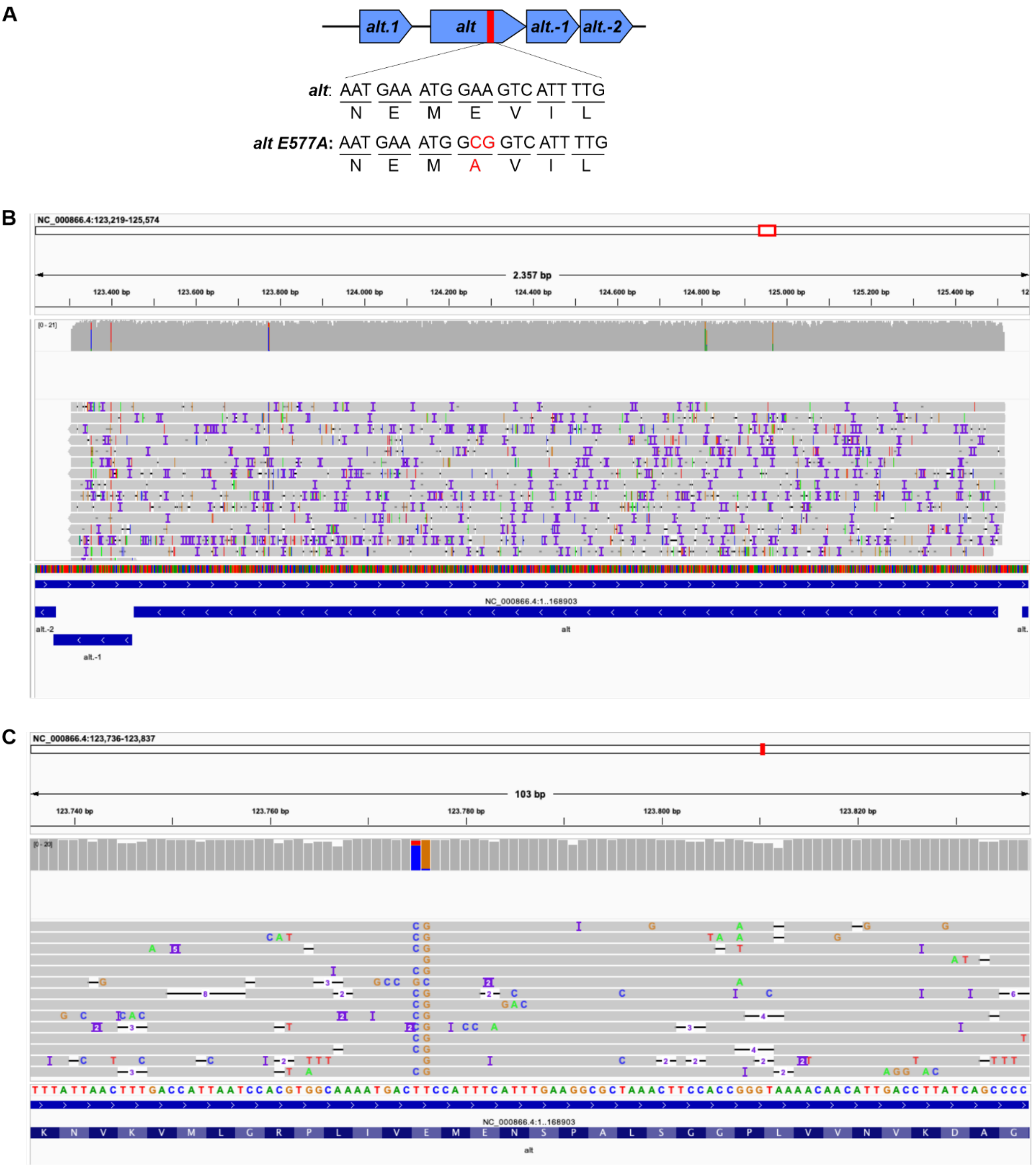

S5 Fig: *Alt* mutants sequencing summary. (**A**) The targeted mutation site in *alt* gene. (**B**) Sequence coverage at the mutation site. (**C**): Alt E577A mutant.
