# Supplementary material for: Temporal epigenome modulation enables efficient bacteriophage engineering and functional analysis of phage DNA modifications
Source: PLoS Genet. 2024 Sep 4;20(9):e1011384. doi: 10.1371/journal.pgen.1011384 (PMC11404850; doi:10.1371/journal.pgen.1011384)
Supplement: S6 Fig — (A) The targeted mutation site in modA gene. (B) Sequence coverage at the mutation site. (C) ModA E165A mutant. (PDF) [file pgen.1011384.s006.pdf]

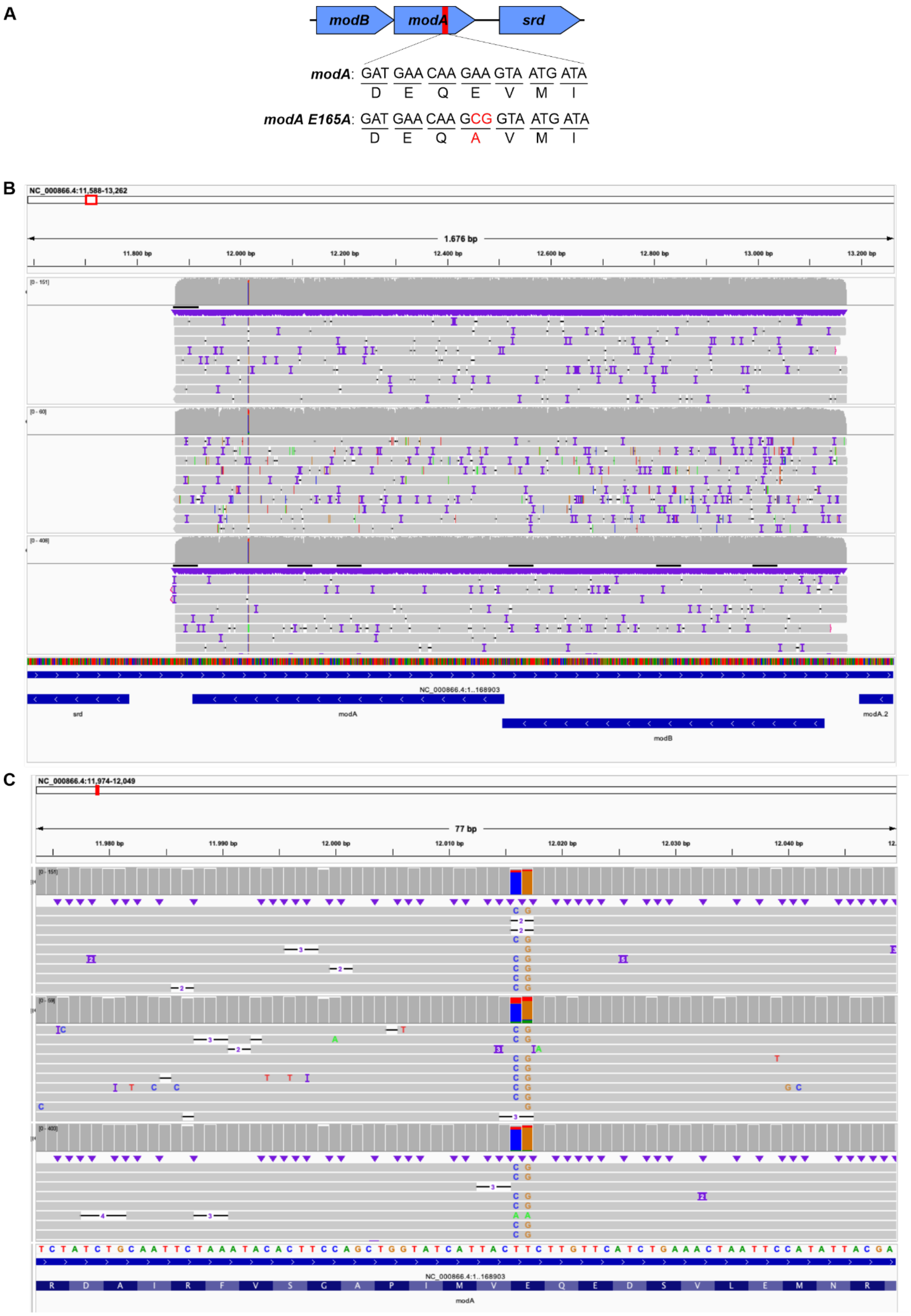

S6 Fig: *ModA* mutants sequencing summary. (**A**) The targeted mutation site in *modA* gene. (**B**) Sequence coverage at the mutation site. (**C**) ModA E165A mutant
